# Effect of dynamic cyclic loading on screw loosening of retightened versus new abutment screw in both narrow and standard implants (in-vitro study)

**DOI:** 10.1186/s40729-020-00229-3

**Published:** 2020-07-28

**Authors:** Eman Mohammed Nasr Attiah, Attiah Ali AlGendy, Tamer Mohamed Nasr Mostafa

**Affiliations:** Prosthodontic Department Faculty of Dentistry, Tanta University, Elgeish St., Tanta, Egypt

**Keywords:** Dynamic cyclic loading DCL, Removal torque loss RTL, Retightening, Screw loosening

## Abstract

**Background:**

The purpose of this in-vitro study was to evaluate the effect of dynamic cyclic loading on screw loosening of retightened abutment screw versus new abutment screw in both narrow and standard implants.

**Methods:**

Separate acrylic resin blocks containing implant assembly (fixture, abutment, abutment screw, metal tube capping the abutment). Samples were divided into two main groups according to the diameter of implant: group 1 (GI 4.5-mm diameter) and group 2 (GII 3-mm diameter). Each group is subdivided into two subgroups according to the suggested option to manage screw loosening either by retightening (GIA, GIIA) or using new screws (GIB, GIIB). One hundred thousand cycles of eccentric dynamic cyclic loading (DCL) were applied before and after retightening or replacing the screw; then, removal torque loss (RTL) ratio was calculated, tabulated, and analyzed by *t*-student, ANOVA, pair wise Tukey’s tests.

**Results:**

There were differences between GI and GII regarding the incidence of screw loosening process. Removal torque loss ratio was higher in GIB and GIIB where the old abutment screws were replaced by new screws for both standard implants (SIs) and narrow diameter implants (NDIs). There was significant effect of retightening and replacing the abutment screws after exposure to DCL.

**Conclusions:**

Within the limitations of this in-vitro study, it can be conclude that screw loosening process occurred in both SIs and NDIs but with higher values in NDIs. It is better to retighten the screw of NDIs and SIs than replacing it with a new screw.

## Introduction

Oral implantology has undergone a well-deserved rebirth or rediscovery and implants considered the treatment of choice in an increasing number of carefully selected cases.

After osseointegration is achieved, long-term clinical follow-ups reported biological or mechanical complications [1]. One of the systematic reviews evaluated the survival rate of implant-supported single crowns concluded that the cumulative incidence of abutment screw or abutment loosening was 7.3% in both external and internal connections from the 26 clinical studies included [2].

Screw loosening may cause implant or screw fracture, inadequate occlusal force distribution, and possible osseointegration failure. In addition, screw loosening would also lead to micro motion at the implant-abutment interface while chewing [3]. Sones [4] reported that the failure of components, especially if abutment screws cannot be retrieved, might necessitate the disuse of the involved implant and require conversion or remake of the prosthesis. Furthermore, granulation tissues between the loose abutment and the implant were found, leading to fistulae formation and infection of the soft tissue [5].

The process of screw loosening was described in two stages [6]. Initially, external forces cause sliding between the threads, partially relieving the stretching of the screw and reducing preload. At this stage, the higher the preload, the greater will be the resistance to loosening. The second stage is attained by a gradual reduction of preload below a critical level, in which external forces cause the turning of the screw in an anti-clockwise direction, and it loses its function. Failures are due to metal fatigue and occur under repeated loads at levels below the maximum strength of the material [7].

Many factors related to screw design and fabrication may affect abutment or prosthetic screw loosening in a metal-to-metal screw system; these primarily are related to preload. Preload can be influenced by component and screw materials [8, 9], torque delivery systems [10], manufacturer’s quality control [11], screw joint design [12], surface roughness [13], and fatigue testing [9, 14]. It was reported that the main factor in screw loosening was an inappropriate tightening torque. If the tightening torque was not consistent, the following preload showed a difference and could affect the removal torque [15].

In addition, factors that affect abutment screws loosening include hex height (or depth), diameter of the screw, platform diameter [16], surface condition, vibrating micro movement, excessive bending [17], microleakage [18], abutment connection [16], abutment diameter [19], surface coating [20], cement wash out [21], collar length [22], abutment angulation [23], lateral cyclic loading [5], inadequate tightening torque, retorque [24], reverse torque [25], and settling effect [26].

One of the variables that influence the joint stability is how the contacting parts change when the screw is tightened. After being tightened together by the screw, the micro-roughness of all the metal contacting surfaces slightly flattens, and the microscopic distance between contacting surfaces decreases [16]. As a result of this process called “settling,” the screw loses part of its preload [26]. For this reason, detorque values immediately after tightening are always lower than the initial tightening torque [7, 27, 28].

When evaluating the screw loosening of new abutment screws and after successive tightening, it was found that the percentage of the initial torque loss is lower when screws that already suffered the application of an initial torque were used, remaining stable after application of successive torques [29] that is why retightening the old screw is a current option.

It was strongly recommended that retightening of implant abutment screw is important to decrease the possible screw loosening. Pardal-Peláez et al. [30] have stated that the most effective strategy is to retighten the screws 10 min after the first tightening, after the 1st year of function and then periodically to compensate the settling effect. Bulaqi et al. [31] have recommended that retightening reduced the settling effect and had an insignificant effect on the preload. At high coefficients of friction, the retightening effect was intensified. Farina et al. [32] concluded that the retorque application significantly increases the loosening torque when titanium and gold screws are used.

If the abutment screw is exposed to excessive wear and still in place, screw replacement is a good option. Hum [33] has introduced a special technique to accurately locate the loose abutment screw and replace it with a new one. Screw replacement may be of damaging effect especially for cement-retained metal-ceramic restorations with ceramic occlusal surfaces. Schwedhelm et al. [34] introduced a technique for locating implant abutment screws of such restorations that may facilitate the clinician’s ability to locate the abutment-screw access in the event of abutment-screw loosening, thus reducing the need for refabricating the restoration.

NDIs are considered a good treatment option replacing missing teeth where there is lack of adequate bone width and interdental space without need for bone augmentation procedures that aim to transform the deficient ridge into a ridge that is capable of receiving conventional tooth-form implants. These advanced augmentation procedures have disadvantages, such as a prolonged healing time, additional cost, and unpredictable complications including infection and wound exposure [35]. Nearly no data available regarding screw loosening of NDIs that is why this study will be carried out.

So the purpose of this study was to evaluate the effect of DCL on screw loosening of retightened abutment screw versus new abutment screw in both NDIs and SIs and to test the null hypothesis that there are no differences between retightening screws and using new abutment screw in both NDIs and SIs.

## Material and methods

Total forty titanium fixtures (B&B Dental Implant Company, Italy) with conical hybrid connection were used in this study and divided into two groups, twenty each according to their diameter and so the diameter of the screw: group I (SIs), implants with 4.5-mm diameter; and group II (NDIs), implants with 3-mm diameter. Each group was divided into two subgroups A and B, ten each according to the suggested solution for screw loosening management, GIA and GIIA for retightening option, and GIB and GIIB for new screw option. For each sample, 12-mm length bone level implants were used with platform switching including conical hybrid connection (Morse Taper 5 degree).

Forty stock straight titanium abutments were selected for this study with the same gingival height of 2 mm and the same post height for standardization. The titanium abutment was screwed to the fixture, sprayed, and *three dimensionally* (3D) scanned to accurately get the desired design and dimensions of the metal tube that fit the abutment head; this was achieved by the *Computer Aided Design Computer Aided Manufacturing* (CAD/CAM) software (Dent create Exocad), with flat occlusal surface (10 mm in diameter) parallel to horizontal plane and perpendicular to implant fixture long axis to permit contact with the testing machine piston in a flat horizontal plane with small rounded hole exactly opposite to the abutment screw hole that facilitates screw driver accessibility. Using a special CAD/CAM wax type, forty identical wax patterns for metal tube of each group were made; these patterns were casted by a lost wax technique. The inner surfaces of the metal tube for each group were checked to the desired dimension using digital caliper and adjusted with a carbide bur, then checked to ensure that each tube fit accurately on corresponding abutment, and the screw driver inserted and removed easily.

Water balance device is adjusted over the top of the metal tube to ensure 180° surface just before cementation to the corresponding abutment; this procedure will be repeated after securing the specimens to the customized jig just before application of dynamic cyclic loading. Self-adhesive resin cement was used for cementation of metal tube to the abutment; the access of screw hole of each abutment was protected by cotton. Finally, all samples had been ready for the testing procedures.

### Recording the initial and post load *removal torque value* (RTV)

**Fig. 1:**
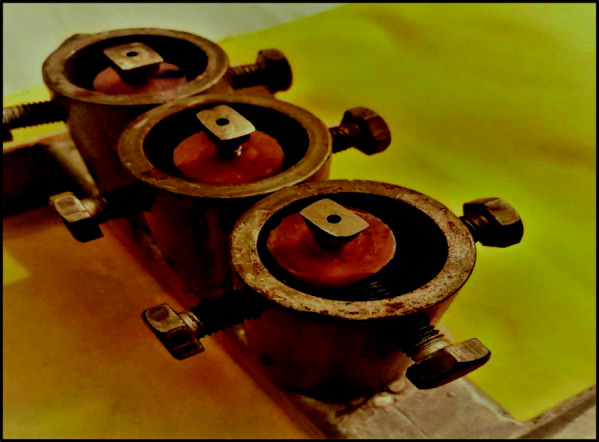
Customized rigid mounting jig with fixating screws and the samples inside

Before measuring the initial RTV, a customized rigid metal mounting jig was used to firmly fix the acrylic sample by tightening three metal screws (Fig. 1).

Abutment screw was tightened to 30 Ncm according to the manufacturer’s recommendation using a digital torque gauge (HTG2- 200Nc, IMADA, Toyohashi, Japan).

Ten minutes after first torque application, a torque of 30 Ncm was reapplied again with the same digital torque gauge. Ten minutes later of screw retightening, the initial RTV was measured by rotating the screw driver in an anticlockwise direction and recorded.

**Fig. 2:**
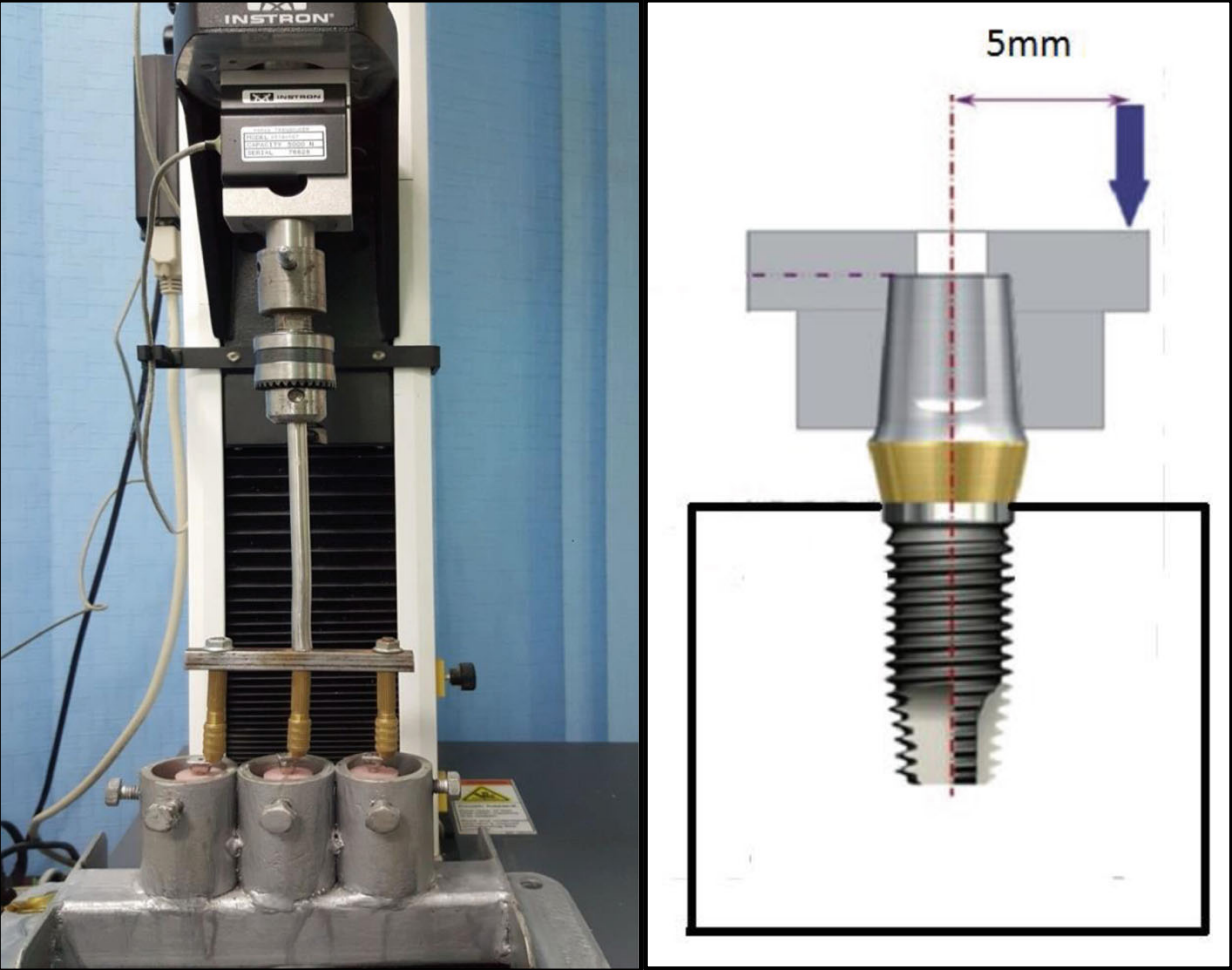
Universal testing machine with samples under dynamic cyclic loading and schematic diagram for testing condition

After measuring initial RTV, the abutment screw was tightened again to the recommended torque value (30 Ncm). Then, immediately the acrylic resin block was firmly mounted in a holder of the lower fixed compartment of a computer controlled universal testing machine (Model 3345; Instron Industrial Products, Norwood, MA, USA), for 100,000 cycles of eccentric dynamic cyclic loading, 5 mm away from the center of the previously fabricated metal tube. The dynamic cyclic loading was performed with a metallic rod with round tip which was attached to the upper movable compartment of the machine, under load of 130 N perpendicular to the metal tube and 5 mm away from the center axis of the implant using a metallic rod with round tip which was attached to the upper movable compartment of the machine. The contact time between the rod and metal tube was adjusted to 0.2 s at a rate of 1 Hz which simulates the tooth contact duration of each masticatory cycle (Fig. 2).

### Calculation of removal torque loss (RTL) ratio of abutment screw before and after dynamic cyclic loading **(***DCL*)

Screw loosening of each assembly (implant-abutment) was analyzed by measuring RTV before and after dynamic cyclic load by using the digital torque gauge. RTL can be an indicator of how much loosening takes place.

Each RTL ratio was calculated using the following formula [14]:

Removal torque loss ratio before loading (% initial RTL)

An indicator of how much loosening takes place before loading.

$$ =\frac{\mathrm{Tightening}\kern0.17em \mathrm{torque}\hbox{-} \mathrm{Removal}\kern0.17em \mathrm{torque}\kern0.17em \mathrm{before}\kern0.17em \mathrm{loading}}{\mathrm{Tightening}\kern0.17em \mathrm{torque}}\times 100 $$

Removal torque loss ratio after loading (% post load RTL)

An indicator of how much loosening occurs after loading.

$$ =\frac{\mathrm{Tightening}\kern0.17em \mathrm{torque}\hbox{-} \mathrm{Removal}\kern0.17em \mathrm{torque}\kern0.17em \mathrm{after}\kern0.17em \mathrm{loading}}{\mathrm{Tightening}\kern0.17em \mathrm{torque}}\times 100 $$

Removal torque loss ratio between before and after loading (% difference between initial and post load RTL).

An indicator of the degree of loosening caused by the DCL.

$$ =\frac{\mathrm{Removal}\kern0.17em \mathrm{torque}\kern0.17em \mathrm{before}\kern0.17em \mathrm{loading}\hbox{-} \mathrm{Removal}\kern0.17em \mathrm{torque}\kern0.17em \mathrm{after}\kern0.17em \mathrm{loading}}{\mathrm{Removal}\kern0.17em \mathrm{torque}\kern0.17em \mathrm{before}\kern0.17em \mathrm{loading}}\times 100 $$

- The screws of specimens of GIA and GIIA were retightened at the same initial torque (30 Ncm) using the digital torque gauge. Then, the procedures of dynamic cyclic loading were repeated for the second time.
- The screws of specimens of GIB and GIIB were changed with new screws, torqued to 30 Ncm, left for 10 min, and torqued again to 30 Ncm, and then the procedures of dynamic cyclic loading were repeated for the second time.
- After DCL, the loading machine was stopped. The acrylic resin block with fixture and abutment was transferred again to the metal jig to measure post load RTV using tire same digital torque gauge with procedures same as measuring initial RTV.

### Statistical analysis

Student’s *t*-test, ANOVA, and pair wise Tukey’s post hoc tests were used to compare mean of initial and post load RTL ratio between different groups and subgroups. Statistical analysis was performed by using SPSS program version 20 (SPSS Inc. Chicago, USA).

## Results

The mean of initial percentage, post load percentage, and percentage of difference between initial and post load removal torque loss after first exposure to dynamic cyclic loading (RTL_1_) was recorded. The mean of initial percentage, post load percentage, and percentage of difference between initial and post load removal torque loss after second exposure to dynamic cyclic loading (RTL_2_) was also recorded for all test groups.

### When comparing standard and narrow implants regarding occurrence of screw loosening process (Tables 1 and 2; Figs. 3 and 4)

#### For standard implants

- Mean percentage initial RTL_1_ was 32.491 ± 0.821 significantly increased to 39.282 ± 2.255.
- Mean percentage difference between initial and post load RTL_1_ was 21.039 ± 8.758.
- Mean initial RTL_2_ was 33.301 ± 11.894 significantly increased to 51.966 ± 3.120.
- Mean percentage difference between initial and post load RTL_2_ was 79.177 ± 72.697.

**Table 1:** Mean ± SD of percentage initial and percentage post load RTL_1_ and percentage difference between initial and post load RTL_1_ for GI and GII (regarding the screw loosening)

% RTL 1		Groups						*T*-test	
		Standard diameter			Narrow diameter			*t*	*P* value
Initial	Range	31.33	−	33.4	20	−	30	8.179	< 0.001*
	Mean ± SD	32.491	±	0.821	24.501	±	2.978		
Post load	Range	36.67	−	42.5	40.33	−	47.67	− 4.488	< 0.001*
	Mean ± SD	39.282	±	2.255	43.766	±	2.213		
Paired test	*P* value	< 0.001*			< 0.001*				
% Difference	Range	10.01	−	35.57	37.78	−	118.33	− 7.398	< 0.001*
	Mean ± SD	21.039	±	8.758	81.110	±	24.139		

*Statistical significant

**Table 2:** Mean ± SD of percentage initial and percentage post load RTL_2_ and percentage difference between initial and post load RTL_2_ for GI and GII (regarding the screw loosening)

% RTL 2		Groups						*T*-test	
		Standard diameter			Narrow diameter			*t*	*P* value
Initial	Range	17.67	−	46.67	21	−	47.67	− 0.063	0.951
	Mean ± SD	33.301	±	11.894	33.633	±	11.763		
Post load	Range	46.67	−	57.33	43.33	−	61	− 0.327	0.747
	Mean ± SD	51.966	±	3.120	52.666	±	6.009		
Paired test	*P* value	0.002*			0.007*				
% Difference	Range	9.37	−	190.57	3.65	−	165.08	− 0.056	0.956
	Mean ± SD	79.177	±	72.697	81.084	±	78.773		

*Statistical significant

**Fig. 3:**
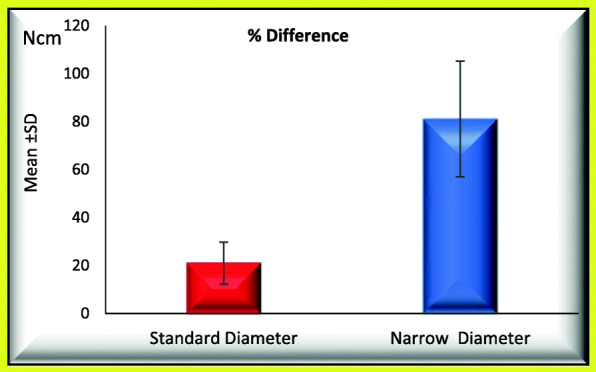
Mean of percentage difference between initial and post load RTL_1_ for GI and GII

**Fig. 4:**
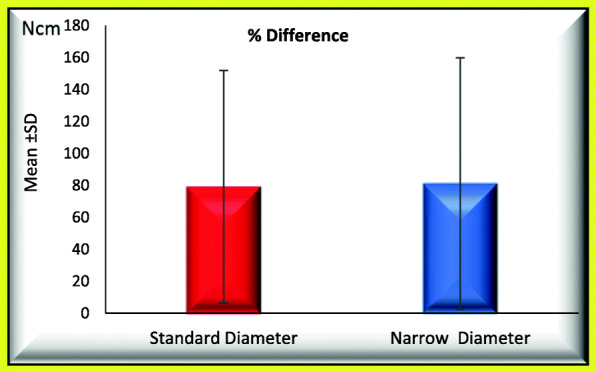
Mean of percentage difference between initial and post load RTL_2_ for GI and GII

#### For narrow diameter, the results were as follows:

- Mean percentage initial RTL_1_ was 24.501 ± 2.978 and significantly increased to 43.766 ± 2.213.
- Mean percentage difference between initial and post load RTL_1_ was 81.110 ± 24.139.
- Mean percentage initial RTL_2_ was 33.633 ± 11.763 significantly increased to 52.666 ± 6.009.
- Mean percentage difference between initial and post load RTL_2_ was 81.084 ± 78.733.

### When comparing standard and narrow implants regarding the retightening option, the following data were found (Figs. 5 and 6)

#### For standard implants (4.5 mm) (Tables 3 and 4)

- Mean percentage initial RTL_1_ was 32.518 ± 0.872 and not significantly increased to 39.362 ± 2.462.
- Mean percentage difference between initial and post load RTL_1_ was 21.192 ± 9.569.
- Mean percentage initial RTL_2_ was 44.402 ± 1.623 and not significantly increased to 49.866 ± 2.408.
- Mean percentage difference between initial and post load RTL_2_ was 12.280 ± 1.975.

**Fig. 5:**
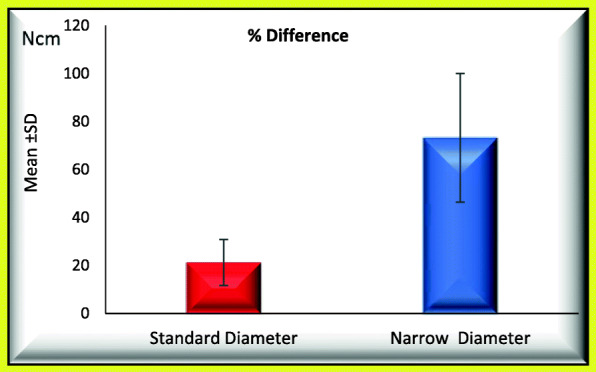
Mean of percentage difference between initial and post load RTL_1_ for GI and GII (regarding retightening option)

**Fig. 6:**
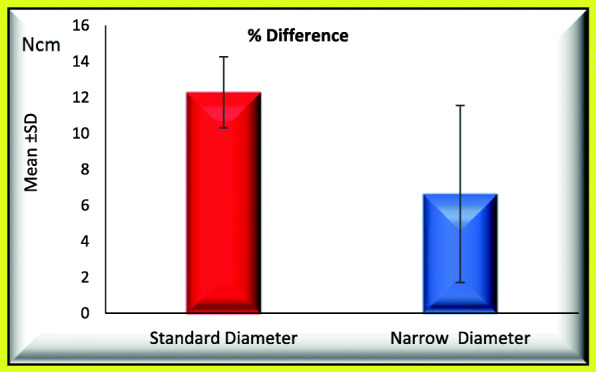
Mean of percentage difference between initial and post load RTL_2_ for GI and GII (regarding retightening option)

**Table 3:** Mean ± SD of percentage initial and percentage post load RTL_1_ and percentage difference between initial and post load RTL_1_ for GIA and GIB

% RTL 1		Standard diameter						*T*-test	
		Retightening			New screw			*t*	*P* value
Initial	Range	31.35	−	33.4	31.33	−	33.33	0.098	0.924
	Mean ± SD	32.518	±	0.872	32.464	±	0.869		
Post load	Range	36.7	−	42.5	36.67	−	42	0.106	0.918
	Mean ± SD	39.362	±	2.462	39.202	±	2.316		
Paired test	*P* value	0.007*			0.006*				
% Difference	Range	10.01	−	35.57	10.02	−	34.06	0.052	0.960
	Mean ± SD	21.192	±	9.569	20.886	±	8.998		

*Statistical significant

**Table 4:** Mean ± SD of percentage initial and percentage post load RTL_2_ and percentage difference between initial and post load RTL_2_ for GIA and GIB

% RTL 2		Standard diameter						*T*-test	
		Retightening			New screw			*t*	*P* value
Initial	Range	42.67	−	46.67	17.67	−	25	15.534	< 0.001*
	Mean ± SD	44.402	±	1.623	22.200	±	2.753		
Post load	Range	46.67	−	53.33	51.33	−	57.33	− 2.848	0.022*
	Mean ± SD	49.866	±	2.408	54.066	±	2.253		
Paired test	*P* value	< 0.001*			< 0.001*				
% Difference	Range	9.37	−	14.29	127.14	−	190.57	− 11.283	< 0.001*
	Mean ± SD	12.280	±	1.975	146.074	±	26.441		

*Statistical significant

#### For narrow diameter implants (3 mm) (Tables 5 and 6)

- Mean percentage initial RTL_1_ was 26.134 ± 2.755 and not significantly increased to 44.666 ± 2.418.
- Mean percentage difference between initial and post load RTL_1_ was 73.160 ± 26.821.
- Mean percentage initial RTL_2_ was 44.666 ± 2.418 and not significantly increased to 47.598 ± 2.734.
- Mean percentage difference between initial and post load RTL_2_ was 6.636 ± 4.913.

**Table 5:** Mean ± SD of percentage initial and percentage post load RTL_1_ and percentage difference between initial and post load RTL_1_ for GIIA and GIIB

% RTL 1		Narrow diameter						*T*-test	
		Retightening			New screw			*t*	*P* value
Initial	Range	23	−	30	20	−	25	2.003	0.080
	Mean ± SD	26.134	±	2.755	22.868	±	2.387		
Post load	Range	41.33	−	47.67	40.33	−	45	1.342	0.216
	Mean ± SD	44.666	±	2.418	42.866	±	1.775		
Paired test	*P* value	0.001*			< 0.001*				
% Difference	Range	37.78	−	107.25	70.42	−	118.33	− 1.047	0.326
	Mean ± SD	73.160	±	26.821	89.060	±	20.825		

*Statistical significant

**Table 6:** Mean ± SD of percentage initial and percentage post load RTL_2_ and percentage difference between initial and post load RTL_2_ for GIIA and GIIB

% RTL 2		Narrow diameter						*T*-test	
		Retightening			New screw			*t*	*P* value
Initial	Range	41.33	−	47.67	21	−	23.67	18.601	< 0.001*
	Mean ± SD	44.666	±	2.418	22.600	±	1.091		
Post load	Range	43.33	−	50	53.67	−	61	− 5.491	0.001*
	Mean ± SD	47.598	±	2.734	57.734	±	3.092		
Paired test	*P* value	0.036*			< 0.001*				
% Difference	Range	3.65	−	15.38	143.94	−	165.08	− 32.431	< 0.001*
	Mean ± SD	6.636	±	4.913	155.532	±	9.014		

*Statistical significant

### When comparing standard and narrow implants regarding use of new abutment screw, the following data were found (Figs. 7 and 8)

#### For standard diameter implants (4.5 mm) (Tables 3 and 4)

- Mean percentage initial RIL_1_ was 32.464 ± 0.869 not significantly increased to 39.202 ± 2.316.
- Mean percentage difference between initial and post load RTL_1_ was 20.886 ± 8.998.
- Mean percentage initial RIL_2_ was 22.200 ± 2.753 and not significantly increased to 54.066 ± 2.253.
- Mean percentage difference between initial and post load RTL_2_ was 146.074 ± 26.441.

**Fig. 7:**
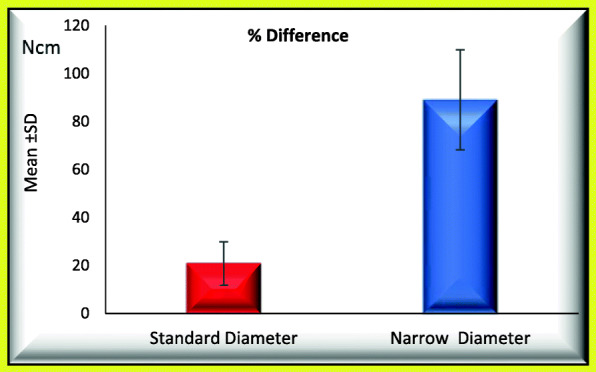
Mean of percentage difference between initial and post load RTL_1_ for GI and GII (regarding use of new abutment screw)

**Fig. 8:**
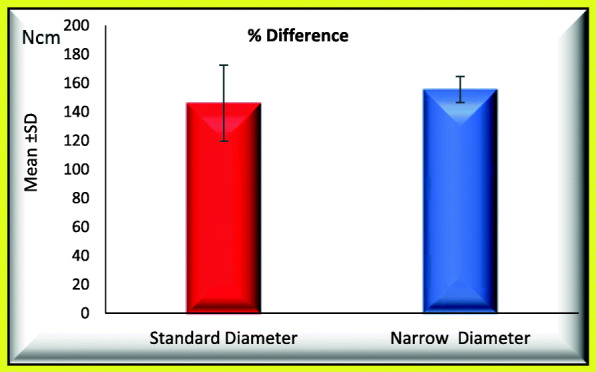
Mean of percentage difference between initial and post load RTL_2_ for GI and GII (regarding use of new abutment screw)

#### For narrow diameter implants (3 mm) (Tables 5 and 6)

- Mean percentage initial RIL_1_ was 22.868 ± 2.387 and not significantly increased to 42.866 ± 1.775.
- Mean percentage difference between initial and post load RTL_1_ was 89.060 ± 20.825.
- Mean percentage initial RIL_2_ was 22.600 ± 1.091 and not significantly increased to 57.734 ± 3.092 (Tables 7 and 8).
- Mean percentage difference between initial and post load RTL_2_ was 155.532 ± 9.014.

**Table 7:** One way ANOVA test comparing between all subgroups regarding percentage initial, percentage post load, and percentage difference between initial and post load (RTL_1_) and percentage initial, percentage post load, and percentage difference between initial and post load (RTL_2_)

		Subgroups												ANOVA	
		GIA			GIB			GIIA			GIIB			*F*	*P* value
Initial; RTL 1%	Range	31.35	−	33.4	31.33	−	33.33	23	−	30	20	−	25	31.153	< 0.001*
	Mean ± SD	32.518	±	0.872	32.464	±	0.869	26.134	±	2.755	22.868	±	2.387		
Post load; RTL 1%	Range	36.7	−	42.5	36.67	−	42	41.33	−	47.67	40.33	−	45	7.097	0.003*
	Mean ± SD	39.362	±	2.462	39.202	±	2.316	44.666	±	2.418	42.866	±	1.775		
Paired test	*P* value	0.007*			0.006*			0.001*			< 0.001*				
% Difference	Range	10.01	−	35.57	10.02	−	34.06	37.78	−-	107.25	70.42	−	118.33	18.784	< 0.001*
	Mean ± SD	21.192	±	9.569	20.886	±	8.998	73.160	±	26.821	89.060	±	20.825		
Initial; RTL 2%	Range	42.67	−	46.67	17.67	−	25	41.33	−	47.67	21	−	23.67	189.380	< 0.001*
	Mean ± SD	44.402	±	1.623	22.200	±	2.753	44.666	±	2.418	22.600	±	1.091		
Post load; RTL 2%	Range	46.67	−	53.33	51.33	−	57.33	43.33	−	50	53.67	−	61	14.492	< 0.001*
	Mean ± SD	49.866	±	2.408	54.066	±	2.253	47.598	±	2.734	57.734	±	3.092		
Paired test	*P* value	< 0.001*			< 0.001*			0.036*			< 0.001*				
% Difference	Range	9.37	−	14.29	127.14	−	190.57	3.65	−	15.38	143.94	−	165.08	165.250	< 0.001*
	Mean ± SD	12.280	±	1.975	146.074	±	26.441	6.636	±	4.913	155.532	±	9.014		

*Statistical significant

**Table 8:** Pair wise Tukey’s post hoc test between all subgroups regarding percentage initial, percentage post load, and percentage difference between initial and post load RTL

Tukey’s test				
		GIA	GIB	GIIA
Initial; RTL 1%	GIB	1.000		
	GIIA	< 0.001*	< 0.001*	
	GIIB	< 0.001*	< 0.001*	0.070
Post load; RTL 1%	GIB	0.999		
	GIIA	0.009*	0.007*	
	GIIA	0.107	0.087	0.600
% Difference	GIB	1.000		
	GIIA	0.002*	0.002*	
	GIIB	< 0.001*	< 0.001*	0.528
Initial; RTL 2%	GIB	< 0.001*		
	GIIA	0.997	< 0.001*	
	GIIB	< 0.001*	0.990	< 0.001*
Post load; RTL 2%	GIB	0.096		
	GIIA	0.542	0.007*	
	GIIB	0.001*	0.167	< 0.001*
% Difference	GIB	< 0.001*		
	GIIA	0.922	< 0.001*	
	GIIB	< 0.001*	0.722	< 0.001*

*Statistical significant

## Discussion

Continuous efforts are exerted to maximize the survival rate of the implant itself, abutment screw, implant abutment connection, and the superstructure and also to minimize the problems that frequently accompany the treatment with dental implants.

The clinician must recognize the possible forces that will be acting on the screw joint so that screw loosening and other possible complications can be minimized or avoided.

The implant system used in this study provides the criteria of having various screw diameters, for standard and narrow implant fixtures; this allows accurate evaluation of the biomechanics of SIs and NDIs [36]. Most of implant systems introduce all implant diameters with the same dimensions of the screw; for this reason, most of NDIs showed many complications when they are tested in many researches.

Implants were placed into acrylic resin as according to De Carvalho et al. [37] who used acrylic resin to be subjected to cyclic loading because acrylic resin has enough flexural strength making it sufficiently tough to allow cyclic testing .Also, its modulus of elasticity (3.4 × 105 lb/in.^2^) is quite close to that of cancellous bone (3.6 × 105 lb/in.^2^).

Conical hybrid connection was selected because it was reported that, among the different internal connection types, the conical hybrid connection showed the best stress distribution as it has a mechanical friction grip that enhance resistance to the lateral forces decreasing the probability of screw loosening, so it was the best connection to be used [38, 39].

The desired design and dimensions of metal tube were designed by the CADCAM software (Dentcreate, Exocad) in wax (CopraDur, White peaks dental solution, Germany), with flat occlusal surface (10 mm in diameter) which was parallel to the horizontal plane and perpendicular to the implant fixture long axis to permit contact with the testing machine piston in a flat horizontal plane. In the center of the flat occlusal surface, a small rounded hole was designed exactly opposite to the abutment screw hole that facilitates screw driver accessibility for easy tightening and removal. Then, this accurately designed wax pattern was casted to a nickel chromium alloy tube [38].

CAD/CAM system was to ensure standardization as CAD/CAM system has the ability to produce physical models using digital methods instead of traditional impression techniques with high error rate, time consuming procedures, and lack of accuracy and standardization [40].

Ten minutes interval was left after first torque application, and all screws were retightened to the same tightening torque (30 Ncm), to compensate for the preload loss due to settling effect of screw thus ensure achieving optimal preload.

Tightening and removal of abutment screws in all the groups was done with a digital torque gauge instead of a hand torque wrench to eliminate the possibility of deviations from exact torque value which gives decimal precision for accuracy and standardization [41, 42]. The samples were placed in a rigid mounting jig to ensure solid fixation without rotation during tightening and removal of the screws [37].

Load was applied eccentrically at a distance of 5 mm away from the center of abutment [43, 44] to simulate lateral component of intraoral forces that have critical effects on joint instability [45, 46]. A better stress distributions when lateral external force components act on the prosthetic abutment.

For all test groups, there were significant differences in RTL ratios before and after application of DCL. These findings emphasize on the occurrence of screw loosening process.

The screws of NDIs are comparable with standard ones because the implant system used in this study provided narrow screws with narrow fixtures and standard screws for standard fixtures on contrast with most of implant systems that provide the same screw diameter for both narrow and standard implants. Standard screw within narrow fixtures (relatively thin walls) makes the force transmission more destructive and shows more biomechanical complications that were reported in most of studies worked on NDIs. This finding is supported by Patterson and Johns [47] who stated that failures are due to metal fatigue and occur under repeated loads at levels below the maximum strength of the material when worked on metal fatigue failure of the gold screw used to retain a fixed prosthesis to Brånemark osseointegrated fixtures/abutments; they emphasized on the necessity of screw design and applying the correct torque to achieve a long fatigue life for the screw.

The results of this study showed that the screw loosening process occurred in both SIs and NDIs with non-significant difference; this can be due to use of various abutment screw diameter. The retained preload inside the standard screw provided more screw stability due to the relative increase in material thickness for the standard screw. In other words, when forces are greater than usual, a larger diameter screw will decrease the risk of loosening or fracture, so the standard screw has superior biomechanics over the narrow one; this finding matches the statements of Byrne et al. [48] who states that, the greater the joint preload, the greater the resistance to loosening, and the more stable the joint that was after using the same screws that were loosened and retightened; tightening was on three occasions to the three insertion torques; they revealed the higher preloads generated using the gold-coated screw with both abutment types; the screw design was the crucial factor not the abutment.

The results of this study revealed that mean percentage initial removal torque loss (% RTL) of NDIs is higher than (% initial RTL) of SIs while mean percentage post load removal torque loss after exposure to dynamic cyclic loading is non-significantly different. This can be explained that the same tightening torque (30 Ncm) is applied for both diameters, so the initial tightening torque is relatively high for NDIs, and this is one of the suggested solutions for decreasing the chance of screw loosening process. This explanation is supported by Siamos et al. [49] who stated that increasing the torque value for abutment screws above 30 Ncm can be beneficial for abutment-implant stability and to decrease screw loosening. On the other hand, Jaarda et al. [50] have concluded that altering the preload torque applied to Nobelpharma gold-retaining screws did not affect their ultimate tensile strength. The ultimate tensile strength of the screws from the two lots used in the study differed, suggesting an unannounced change in component specifications. The mechanical integrity of the abutment/implant system depends on two factors: the contact area between the components and the screw’s effectiveness [51]. When two metal surfaces are in contact, adhesion and friction forces do limit the movement between them. When there is full contact between the surfaces, elongation properties of the screw will increase loosening resistance due to higher contact forces over the screw [52].

Another study has shown that retorque does not significantly interfere on the loosening torque when the titanium screws are used in dentures with passive fit. On the other hand, the retorque significantly increased the loosening torque when these screws were used in dentures with misfit [28].

Replacing the old abutment screw with new one showed better results in SIs than NDIs with non-significant difference after two exposures of DCL; this is due that retaining much preload within standard screws will in turn increase the clamping force between screw threads and internal threads of screw channel making it more liable to surface flattening and excessive wear that is considered actual screw failure and requires its replacement with new functioning one. This finding is supported by Haak et al. [7] who discussed the elongation and preload stress in dental implant abutment screws and concluded that tightening the screws beyond recommended levels may be beneficial without producing plastic deformation.

Other authors confined replacing the old screw with new one to the fractured abutment screw that no more performs function under loading. Fracture of the implant abutment screw can be a serious problem, as the fragment remaining inside the implant may prevent the implant from functioning efficiently as an anchor.

Flanagan [53] had introduced many cases of fractured abutment screw and also introduced a technique for abutment, fragment retrieval, crown-abutment separation, crown recementation, and over denture retainer fracture. Nergiz et al. [54] had also introduced a clinical method of removal of a fractured implant abutment screw with successful utilization of the existing prosthesis. Reyhanian et al. [55] also managed the fractured abutment screw with replacing it by new one with care to avoid any fracture of implant abutments and to use the repair kits offered by some implant systems, such as ITI® Dental Implant System (Institut Straumann AG, Switzerland), consists of drills, two drill guides, and six manual tapping instruments, IMZ® (TwinPlus Implant System DENTSPLY Friadent, Germany), only in exceptional circumstances.

This finding is supported by Barbosa et al. [29] who evaluated the screw loosening on new abutment screws and after successive tightening and concluded that loosening percentage of the initial torque is smaller when using screws that already suffered application of an initial torque staying stable after successive tightening procedure. This sign was proven by the SEM (Scanning Electron Microscopic) analysis, which showed removal of the screws spirals irregularities after successive torques. Such event could explain why the values of detorque increased after the second detorque, and the samples remained constant in the subsequent detorques in all body tests. The removal of the surface irregularities must allow less friction between the screw surface and the internal implant surface, favoring the screw sliding and a higher preload transmission.

But this finding is opposed by Bacchi et al. [56] who stated that use of conventional titanium screws for fixation of universal abutments provides higher loosening torque values even after application of DCL, irrespective of the technique applied. The suggested reason for this was that the application of a longer torque period or retorque once again after embedment relaxation or settling would act to regain preload and to increase contact area between the threads. This study evaluated full arch prostheses supported by five implants. Full-arch prostheses are more likely to dissipate cyclic loading along all components, reducing the effect of loads on the screws in comparison to single-crowns.

Another study has shown that retorque does not significantly interfere on the loosening torque when the titanium screws are used in dentures with passive fit. On the other hand, the retorque significantly increased the loosening torque when these screws were used in dentures with misfit [28].

## Conclusions

Within the limitations of this in-vitro study, it can be concluded as follows:

- Screw loosening process occurred in both SIs and NDIs, but it had higher values in NDIs.
- Higher RTVs are obtained after retightening the old abutment screws of both SIs and NDIs after exposure to 100,000 cycles of DCL, in comparison with replacing the old screw with new one.
- Higher RTVs are obtained after replacing the old screws with new ones of SIs after exposure to 100,000 cycles of DCL, in comparison with NDIs.

## Data Availability

Authors declare that they have full control on all data and materials of this study.

## Abbreviations

RTV: Removal torque value
RTL: Removal torque loss
CAD CAM: Computer Aided Design Computer Aided Manufacturing
DCL: Dynamic cyclic loading
3D: Three dimensional
NDIs: Narrow diameter implants
SIs: Standard implants
